# Obstructive Sleep Apnea and Hypertensive Heart Disease: From Pathophysiology to Therapeutics

**DOI:** 10.31083/j.rcm2412342

**Published:** 2023-12-06

**Authors:** Lu Liu, Yi Wang, Liqiong Hong, Nicola Luigi Bragazzi, Haijiang Dai, Huimin Chen

**Affiliations:** ^1^Department of Cardiology, The Second Affiliated Hospital, Zhejiang University School of Medicine, 310000 Hangzhou, Zhejiang, China; ^2^Human Nutrition Unit, Department of Food and Drugs, Medical School, University of Parma, 43125 Parma, Italy

**Keywords:** obstructive sleep apnea, hypertensive heart disease, pathophysiology, therapeutics

## Abstract

Obstructive sleep apnea (OSA) is characterized by recurrent episodes of complete or partial obstruction of the upper airway that lead to intermittent hypoxemia, negative intrathoracic pressure, hypercapnia, and sleep disturbances. While OSA is recognized as a significant risk factor for cardiovascular disease, it’s relationship with hypertensive heart disease (HHD) remains underappreciated. HHD is a condition characterized by the pathological hypertrophy of the left ventricle, a consequence of elevated arterial hypertension. Interestingly, both OSA and HHD share similar underlying mechanisms including hypertension, left ventricular hypertrophy, myocardial fibrosis, oxidative stress, and inflammation, which ultimately contribute to the progression of HHD. This review aims to shed light on the potential role of OSA in HHD pathogenesis, summarizing current OSA treatment options. It is hoped that this review will encourage a renewed clinical focus on HHD and underscore the need for further OSA research, particularly in the context of screening and treating HHD patients.

## 1. Introduction

Hypertensive heart disease (HHD) is characterized by pathological hypertrophy of the left ventricle due to arterial hypertension, leading to extensive changes in heart muscle structure. This remodeling encompasses both cardiomyocyte growth and changes in other cell types within the myocardium [1]. As of 2017, HHD’s worldwide age-standardized prevalence was 217.9 per 100,000—a 7.4% increase since 1990 [2]. HHD is the second leading cause of heart failure (26.2%), trailing only ischemic heart disease (26.5%) [3]. Thus, HHD has become a major cause of global morbidity and mortality [2]. Given its significant impact on global health, it’s essential to identify and address any treatable contributing factors, particularly obstructive sleep apnea (OSA).

OSA is an increasingly common disorder characterized by repeated upper airway collapse, resulting in intermittent hypoxia, increased negative intrathoracic pressure, elevated sympathetic nervous system activity (SNA), increased blood pressure, and frequent sleep disturbances [4, 5]. OSA may also precipitate anxiety, depression, and other emotional disturbances [6] that can adversely affect cardiovascular structure and function [7]. Furthermore, OSA is associated with a range of cardiovascular complications, encompassing hypertension [8], coronary artery disease [9], stroke [10], heart failure [11], and more [12]. In the context of HHD, it is essential to explore the potential pathophysiological connections between OSA and the progression of HHD. This review aims to highlight the role of OSA in HDD pathophysiology and to discuss the impact of OSA treatments on HHD prognosis. This underscores the importance of early OSA detection and treatment in patients with HHD.

## 2. Epidemiology of OSA and HHD

The global prevalence of OSA is rising sharply, affecting almost 1 billion people worldwide [13]. In the U.S., the Wisconsin sleep cohort study found that among adults aged 30 to 70, at least mild OSA is present in 17.4% of women and 33.9% of men while moderate or severe OSA is present in 5.6% of women and 13.0% of men [14]. The study also revealed a 30% surge in OSA cases between 1990 and 2010, translating to a 4.2% rise among women and 7.5% rise among men [14]. It has emerged as a significant risk factor for a variety of cardiovascular disorders, either as the primary cause or as an aggravating factor, including its potential impact on HHD [15]. Other studies have confirmed that HHD is on the rise, with a 7.4% global increase since 1990, reaching 17.1 million cases in 2017 [2]. As HHD is recognized as a condition associated with aging, its impact on both individuals and the overall disease burden escalates with the aging of patients and the population, respectively [16].

Although direct data on the incidence of OSA in HHD is limited, insights can be inferred by examining its prevalence in other cardiovascular conditions. The association between OSA and hypertension has been extensively investigated, with OSA prevalence in hypertensive patients ranging from 40% to 80% [17]. Several studies have also indicated a connection between OSA and left ventricular hypertrophy (LVH). For example, Cloward *et al*. [18] utilized echocardiography to observe LVH in 22 out of 25 patients (88%) with severe OSA. Meanwhile, by Sukhija *et al*. [19] reported echocardiographic LVH in 78% of patients with moderate to severe OSA, 46% with mild OSA, and 23% without OSA. Among OSA patients, atrial fibrillation is prevalent in 5% [20], while it is worth noting that the prevalence of OSA among individuals diagnosed with atrial fibrillation has been reported as high as 32% to 39% [21]. Sleep apnea exhibits a notably high prevalence among patients with heart failure, with studies indicating rates ranging between 50% and 70% [22]. It’s worth mentioning that OSA constitutes around one-third of the documented sleep apnea cases in this population [11]. Future findings are expected to provide direct evidence on the incidence of OSA in patients with HHD.

## 3. Screening and Diagnosis of OSA

**Fig. 1.**
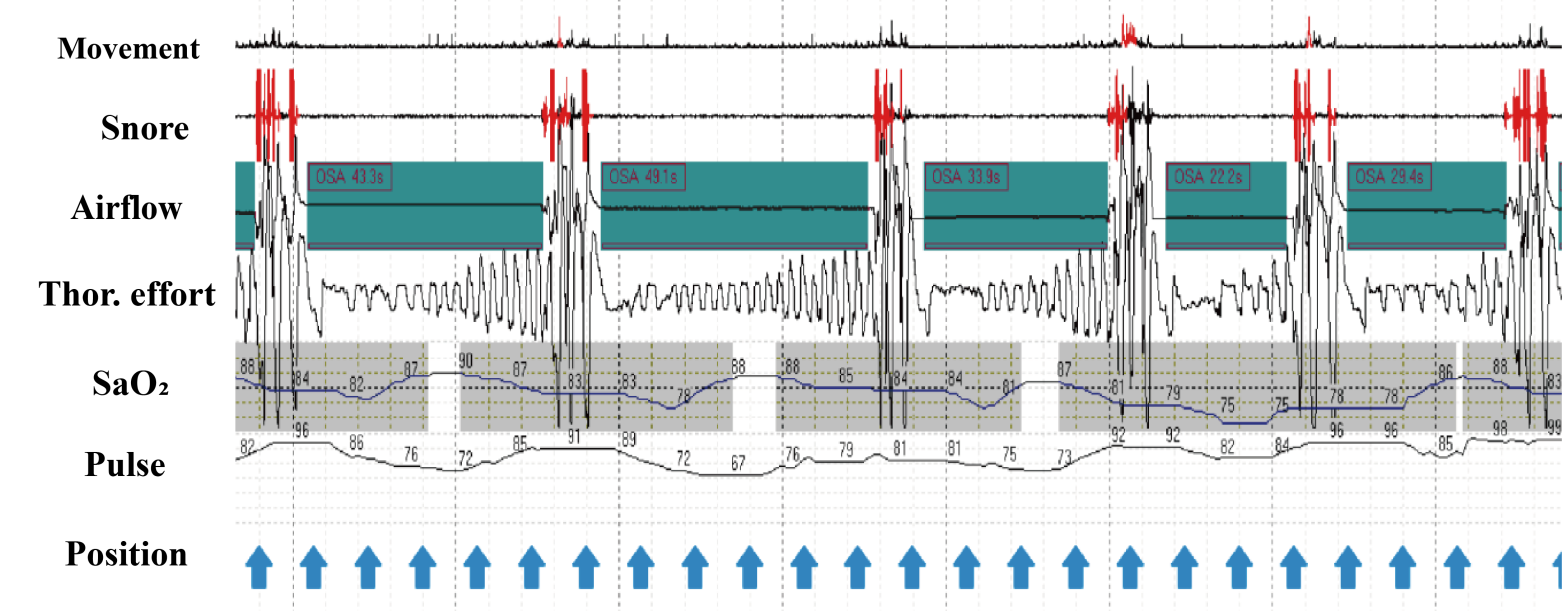
**Characteristic home sleep apnea testing traces**. During each obstructive apnea, the absence of airflow is concurrent with thoracic effort. Snoring occurs irregularly and intermittently due to apnea; ${\mathrm{SaO}}_{2}$ decreased and delayed relative to the apnea. OSA, obstructive sleep apnea.

Given the elevated occurrence of OSA and the tendency of patients to not always report sleep problems to clinicians, screening is of paramount importance. OSA screening typically entails the use of questionnaires that assess OSA symptoms and may additionally consider clinical factors such as body mass index, neck circumference, and blood pressure [23]. Examples of screening questionnaires encompass the Epworth sleepiness scale, STOP-bang (snoring, tiredness, observed apnea, blood pressure, body mass index, age, neck circumference, and gender) questionnaire, STOP questionnaire, the Berlin questionnaire, the Wisconsin sleep questionnaire, and the multivariable apnea prediction tool [12]. If the answers to a screening questionnaire suggest the potential presence of OSA, it is common practice to recommend a diagnostic sleep study. The diagnosis of OSA typically involves overnight polysomnography performed in a specialized sleep laboratory. This diagnostic examination monitors various parameters, including sleep patterns, airflow, cardiac rhythm, thoracoabdominal movements and oxygen saturation levels (${\mathrm{SaO}}_{2}$) [24]. However, due to limited accessibility to polysomnography, home sleep apnea testing has been developed for convenient in-home screening and diagnosis of OSA, and includes measures such as snoring, airflow, respiratory effort, and oxygen saturation, etc., as shown in Fig. 1 [25].

Apnea is clinically characterized by a substantial reduction in tidal volume, often reaching up to 90%, and lasting for at least 10 seconds, typically accompanied by a 3% decrease in oxygen saturation (${\mathrm{SaO}}_{2}$) or interrupted by an arousal from sleep [26]. The severity of OSA is determined by the apnea-hypopnea index (AHI) or respiratory event index [27]. The empirical severity is classified into three categories based on the following ranges: 5 to <15 (mild), 15 to 30 (moderate), and >30 (severe) [28].

## 4. Pathophysiological Mechanisms of OSA that Promote HHD

**Fig. 2.**
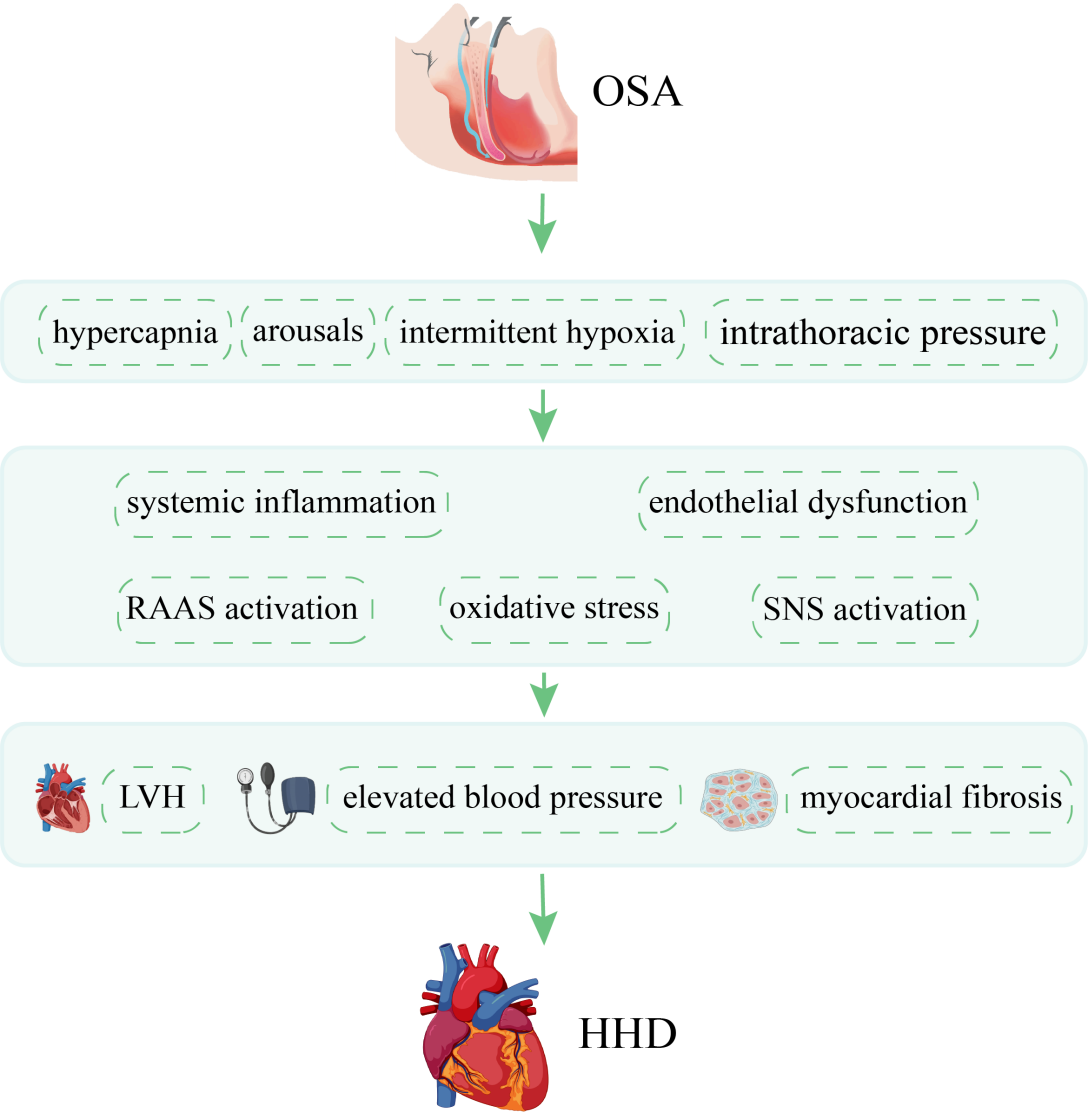
**Pathophysiology of OSA in HHD **. This diagram illustrates a number of mechanisms by which OSA promote HHD. OSA, obstructive sleep apnea; HHD, hypertensive heart disease; RAAS, renin-angiotensin-aldosterone system; SNS, sympathetic nervous system; LVH, left ventricular hypertrophy.

The pathophysiology of HHD is complex and involves the interplay of many mechanisms, including pressure overload, neurohormonal activation, and inflammation. Prolonged exposure to elevated blood pressure leads to LVH, which is the primary compensatory mechanism to maintain cardiac output [29]. However, persistent LVH impairs diastolic function and increases myocardial oxygen demand, leading to ischemia and fibrosis [30]. Additionally, hypertension-induced endothelial dysfunction, oxidative stress, and activation of the renin-angiotensin-aldosterone system (RAAS) promote myocardial inflammation, fibrosis, and apoptosis, further exacerbating cardiac dysfunction [31, 32]. Overall, the cardiovascular effects of OSA mirror those of HHD, potentially exacerbating its onset and progression (Fig. 2).

While the clinical diagnosis and treatment for OSA have become standardized and advanced rapidly, a greater emphasis should be placed on comprehending the core molecular mechanisms driving OSA pathogenesis in the context of treatment, with a goal of either preempting or curing OSA [33]. Certain miRNAs that are either up-regulated or down-regulated by intermittent hypoxemia (IH) serve as direct targets of hypoxia-inducible factor (HIF)-1α, HIF-2α, nuclear factor κB (NF-κB), or their responsive genes, as well as certain inflammatory signaling pathways [34, 35, 36, 37]. We are encouraged to identify miRNAs with differential expression and explore their potential roles or locations. The alterations, whether up-regulated or down-regulated, in long non-coding RNAs induced by chronic rat models also furnish us with valuable insights into potential targeted therapies [38]. Moreover, small molecular compounds play a crucial role in both regulating OSA and advancing mechanistic research. Melatonin, a hormone involved in sleep regulation, confers a variety of advantages, such as serving as a potent antioxidant, decreasing chemoreflex sensitivity, stabilizing ventilatory control, and alleviating the severity of OSA [39]. When it comes to medication, it’s interesting to find that some antihypertensive drugs can reduce the severity of OSA, particularly diuretics [40]. This could be related to diuretics effectively improving fluid overload [41].

### 4.1 The Effect of OSA on Blood Pressure

Hypertension in individuals with OSA arises from a multitude of interlinked pathophysiologic mechanisms. Recurrent apnea leads to IH, sleep fragmentation, and other factors, all of which induce a range of autonomic, hemodynamic, and biochemical changes that contribute to elevated blood pressure. Hypoxia stimulates carotid chemoreceptors and aortic bodies, leading to heightened sympathetic activity [42]. Endothelial dysfunction, another factor in OSA-related hypertension, inhibits the production of nitric oxide (NO), resulting in reduced vasodilation and increased vasoconstriction [43]. Moreover, oxidative stress from OSA generates reactive oxygen species that further inhibit NO synthesis, promote endothelin-1 production, and activate angiotensin II, which collectively contribute to vasoconstriction [44, 45, 46]. Furthermore, OSA induced systemic inflammation worsens endothelial dysfunction [47, 48], while activation of the RAAS raises plasma aldosterone levels, amplifying hypertension [49].

### 4.2 The Effect of OSA on LVH

Longitudinal studies have consistently identified hypertension as the primary factor predisposing patients to LVH [50]. Interestingly, OSA has emerged as an independent LVH trigger, even after accounting for blood pressure levels [51, 52]. A Japanese study specifically investigated the association between OSA and LVH, independently confirming the relationship [53]. In a separate study analyzing 150 newly-diagnosed, untreated OSA patients, multivariate analysis identified autonomous connections between clinic systolic blood pressure and the average nocturnal oxygen saturation, each having an association with LVH [54]. These results have been corroborated in other studies [55, 56, 57]. Generally, the severity of OSA is directly proportional to the prevalence of LVH [58]. The elevated occurrence of LVH in OSA patients largely stems from increased left ventricular afterload and heightened sympathetic activity during apnea episodes [59]. Furthermore, chronic IH in OSA is a contributing factor to LVH. In a rat study, it was demonstrated that left ventricular dysfunction is correlated with myocyte hypertrophy and apoptosis in response to hypoxia [56]. Mouse studies also provide evidence suggesting that LVH is caused by OSA-induced oxygen deprivation [60]. Thus, IH is considered fundamental to the development of left ventricular (LV) dysfunction in OSA [61]. Additional factors thought to play a role in LVH among OSA patients encompass periodic negative intrathoracic pressure overloads affecting the ventricular wall and nocturnal blood pressure surges during sleep [62].

### 4.3 The Effect of OSA on Myocardial Fibrosis

Within the context of HHD, cardiac fibrosis plays a crucial role and defined by the accumulation of extracellular matrix (ECM) proteins, both in the myocardium and adjacent microvasculature [63, 64]. An essential milestone in fibrosis development involves the activation of resident cardiac fibroblasts, leading to their differentiation into myofibroblasts [65, 66]. Myofibroblasts contribute to increased deposition of ECM proteins while concurrently suppressing matrix degradation pathways [67]. Additionally, cardiac fibrosis entails the activation of neurohormonal pathways such as the RAAS, adrenergic signaling, growth factors, cytokines, reactive oxygen species (ROS), and intracellular signaling mechanisms. These factors synergistically modulate ECM remodeling and the formation of fibrous tissue [68, 69]. Numerous clinical and experimental studies have demonstrated a link between OSA and myocardial fibrosis [70, 71, 72]. Increases in the severity of OSA have been independently associated with more severe myocardial fibrosis [73]. Additionally, OSA-induced negative intrathoracic pressure may impair diastolic function, which is further associated with myocardial fibrosis [74]. One of the main pathological factors, IH, elevates plasma levels of thrombospondin-1, which activates transforming growth factor-beta signaling and promotes the transformation of cardiac fibroblasts into myofibroblasts [75]. Another study discovered that IH-induced cardiac fibrosis was associated with increased activity of sodium-hydrogen exchanger-1 in cardiac cells [76]. Moreover, chronic IH has been found to promote myocardial fibrosis in rats [77]. A systematic review also supports these findings [78]. Additionally, activation of the RAAS, another consequence of OSA, can further promote myocardial fibrosis [79].

### 4.4 The Effect of OSA on Oxidative Stress and Inflammation

The growing body of data underscores the influence of non-hemodynamic factors in the pathophysiology of HHD, including changes in oxidative balance and immune responses [80, 81]. In the context of IH, oxidative stress is thought to arise from diminished antioxidant defenses during hypoxic periods and increased reactive oxygen species (ROS) production during reoxygenation, a pattern resembling ischemia-reperfusion injury [47]. Comparative studies have shown that monocytes and granulocytes from individuals with OSA exhibit higher levels of ROS production compared to control subjects, highlighting the role of oxidative stress in this condition [47]. Recent advancements in breath analysis have further identified the presence of a family of compounds associated with oxidative stress in breath samples from OSA patients [47]. Additionally, the periods of hypoxia trigger an inflammatory response involving various proinflammatory cytokines and growth factors that can impact the myocardium in multiple ways. In a rat model, Chen *et al*. [82] observed an increase in LV mass characterized by eccentric hypertrophy following 8 weeks of IH. Furthermore, significant elevations in tumor necrosis factor-α, interleukin-6, insulin-like growth factor, signal transducers and activators of transcription (STAT)-1 and STAT-3, phosphorylated p38 mitogen-activated protein kinase, mitogen-activated protein kinase, and extracellular signal-regulated kinase were observed in cardiac tissue compared to the non-hypoxia group [82].

## 5. Treatment Options for OSA

While limited research exists on the direct impact of OSA treatments on HHD, evidence suggests potential benefits, particularly given the positive outcomes for managing hypertension and enhancing left ventricular diastolic function. Primary treatment modalities for OSA encompass medical devices, behavioral interventions, and surgical options.

### 5.1 Continuous Positive Airway Pressure

Continuous positive airway pressure (CPAP) has firmly established itself as the gold standard first-line treatment for moderate to severe OSA, effectively normalizing the AHI in over 90% of patients using the device [83, 84]. Specifically, in individuals with resistant hypertension and OSA, a 3-month CPAP treatment regimen significantly reduced daytime diastolic blood pressure and 24-hour systolic and diastolic blood pressure, particularly when CPAP adherence exceeded 5.8 hours per night [85]. Additionally, Shim *et al*. [86] conducted a study demonstrating the improvement of left ventricular diastolic function, following three months of CPAP therapy when compared to sham treatment. In a study by Butt *et al*. [87], CPAP therapy was shown to reduce posterior wall thickness and enhance cardiac parameters including LV ejection fraction, systolic velocity, as well markers of diastolic LV impairment. While CPAP has demonstrated its efficacy in reducing blood pressure among hypertensive patients, its long-term impact on cardiovascular events remains inconclusive [88, 89]. In a randomized trial involving more than 2000 patients with established cardiovascular or cerebrovascular disease, participants were assigned to receive either CPAP in addition to their usual care or usual care alone [90]. Over an average 43-month follow-up, CPAP treatment did not significantly reduce the primary composite endpoint of major adverse cardiovascular events [90]. However, a pre-specified subgroup analysis revealed that patients maintaining CPAP for over 4 hours per night exhibited a decreased risk of stroke (hazard ratio [HR], 0.56; 95% confidence interval [CI], 0.32–1.00) and total cerebrovascular events (HR, 0.52; 95% CI, 0.30–0.90) [90].

### 5.2 Lifestyle Modification

Lifestyle modification aimed at managing sleep apnea include weight loss, avoiding sleeping in a supine position, engaging in regular aerobic exercise [26]. Weight loss is particularly impactful, serving as an initial treatment option for patients with minimal symptoms, especially when overweight or obese, and can be combined with other therapies [91]. In a longitudinal study that included individuals within the normal weight range, a 10% increase in weight was associated with a six-fold rise in the risk of developing OSA, whereas a 10% reduction in weight led to a substantial 26% decrease in the AHI [92]. Furthermore, weight loss has the potential to improve blood pressure [93]. Notably, in obese individuals, weight loss led to more significant regression of LVH compared to those of normal weight, irrespective of blood pressure [94]. This suggests that weight reduction has a greater influence on left ventricular mass than hemodynamic factors alone. In a cohort comprising overweight, sedentary men and women, the combination of exercise and weight loss led to a reduction in blood pressure and brought about favorable alterations in left ventricular structure [95]. Exercise may have a positive impact on OSA regardless of weight loss, and there is a dose-dependent correlation between exercise and a reduced prevalence of OSA [96, 97, 98]. Positional therapy is appropriate for individuals with positional OSA. It is estimated that over half of all patients with an AHI exceeding 5 events per hour exhibit a positional component as a contributing factor to their sleep apnea [99]. The impact of one-month positional therapy on 24-hour blood pressure was evaluated in a group of 13 patients with positional OSA, among both hypertensive and normotensive patients, a significant reduction was observed in mean 24-hour blood pressure [100].

### 5.3 Surgery

The evidence supporting surgical interventions for OSA is inconclusive due to the variety of procedures performed, the lack of cardiovascular endpoint measurements, and the infrequent use of long-term follow-up assessments [101]. Surgical modification of the upper airway is a viable option for specific patients and is frequently suggested for symptomatic individuals who find it challenging to tolerate CPAP therapy [102]. An emerging surgical technique, barbed reposition pharyngoplasty, shows promise in alleviating OSA [103]. According to a review, sleep surgery performed on patients with OSA leads to a notable improvement in OSA, as evidenced by a substantial decrease in AHI by 26.2 events per hour. Additionally, there is a significant reduction in office systolic blood pressure by 5.6 mmHg and a significant decrease in diastolic blood pressure by 3.9 mmHg [104]. Result from Seyed Resuli’s research supports this conclusion. Hypertensive patients showed preoperative systolic and diastolic blood pressures averaging 152.3 ± 21.2 and 97.2 ± 12.3 mmHg [105]. Post-uvulopalatopharyngoplasty, these averages dropped to 124.5 ± 19.3 and 86.4 ± 12.1 mmHg, respectively, a statistically significant change (*p* < 0.05) [105].

## 6. Conclusions

Both OSA and HHD are very common globally and place a huge burden on society. The impact of OSA on various cardiovascular diseases is well-established, yet its effect on HHD remains unknown. Interestingly, we observed significant parallels in the pathophysiology of OSA and HHD. Pathophysiology of OSA is linked to the activation of a diverse array of inflammatory, metabolic, neural, and hemodynamic changes, which can have an impact on left ventricular structure and function [31]. The pathophysiology of HHD has been extensively studied, focusing on the widely discussed mechanisms of LVH resulting from hypertension, as well as myocardial fibrosis [30]. Substantial evidence suggests that the pathology of OSA may contribute to the development of HHD. In this review we have summarized the pathophysiological evidence linking the two diseases, providing a new focus on OSA for the clinical management of HHD. We summarized the common clinical treatment of sleep apnea, to provide suitable choice for patients with HHD.

Although OSA is a well-known contributor to cardiovascular disease, there is a surprising lack of research exploring whether OSA treatments lead to reduced cardiovascular events [101, 106]. We can’t seem to get an accurate conclusion at present. One challenge when conducting randomized controlled trials (RCTs) focused on cardiac outcomes in individuals with OSA is the ethical dilemma that frequently arises. This ethical dilemma often leads to the exclusion of individuals experiencing severe sleepiness or hypoxemia, precisely the groups that stand to derive the greatest potential benefits from therapeutic interventions [106]. In addition, treatment adherence needs to be considered. Future studies are needed to assess the effect of OSA treatment on HHD endpoints to provide us with accurate conclusions.
